# Impact of KDO in biological activity of Re-LPS

**DOI:** 10.1186/cc14012

**Published:** 2014-12-03

**Authors:** I Prokhorenko, S Zubova, D Kabanov, S Grachev

**Affiliations:** 1 Institute of Basic Biological Problems, Pushchino, Moscow Region, Russia

## Introduction

The minimal biological active structure of endotoxins (lipopolysaccharides (LPS)) is Re-LPS (KDO2-lipid A), which consist of lipid A and two (or three) molecules of 3-deoxy-D-manno-2-octulosonic acid (KDO) [1,2]. Biological activity of endotoxins is defined in general by the number and distribution of acyl residues on the lipid A backbone [3]. Recently it has been reported that KDO-treated RAW 264.7 cells exhibited a gene expression pattern similar to that in LPS-treated cells. These authors revealed that free KDO participated in crosstalk between Toll-like receptors (TLR) and G protein-coupled receptors and so that regulated activators and repressors of immune signaling [4]. LPS-dependent TLR4-triggered activation of target cells leads to specific changes in the levels of surface receptors and induces synthesis of proinflammatory cytokines [5]. However, the dependence of these processes on the structural composition of LPS is not well understood. To extend our knowledge in this field, the effects of free KDO as well as KDO as covalently linked to lipid A constituent of Re-LPS on expression of TLR4, CD11b and CD14 receptors and TNFα synthesis in whole human blood have been investigated.

## Methods

Human blood was incubated with Re-LPS from *Escherichia coli *JM103 or *Salmonella enterica sv Typhimurium *SL1181 (100 ng/ml) or with lipids A from *E. coli *F583 or *S. enterica *sv Minnesota R595 (80 ng/ml) or with ammonium salt of KDO (20 ng/ml) at 37°C in 5% CO_2_-humidified atmosphere for 2 or 6 hours to determine receptor expression or TNFα release, respectively. Receptor expression was monitored by EPICS XL-MCL flow cytometer using Alexa Fluor 488 anti-TLR4 (HTA125), anti-CD11b (ICRF44) and anti-CD14 (HCD14) antibodies. Human TNF-α ELISA Kit II was exploited to TNFα determination.

## Results

**Figure 1:**
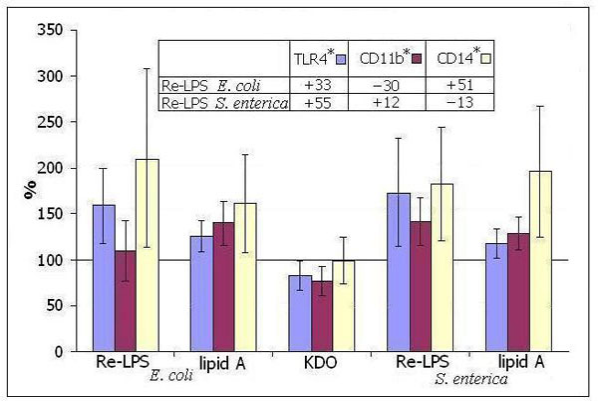
**Expression of TLR4, CD11b and CD14 on monocytes after incubation of whole blood with Re-LPS, lipid A or KDO**. Presented are the results of six independent experiments. Alteration in receptor expression was calculated according to the control level that had been expressed as 100%. *Changes in receptor expression were calculated as %MnIX [KDO2-lipid A] - %MnIX [lipid A].

**Figure 2:**
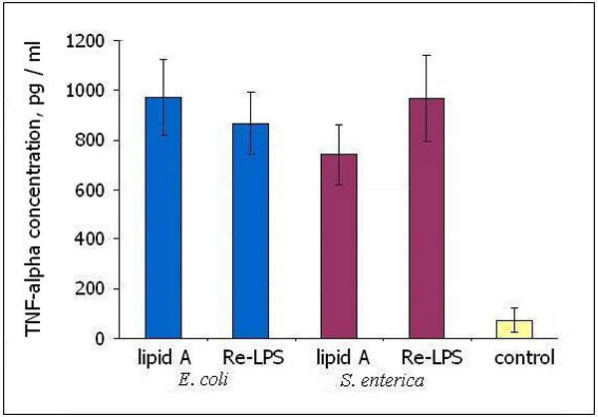
**Production of TNFα after incubation of whole blood with lipid A or Re-LPS**.

Re-LPS *E. coli *or Re-LPS *S. enterica *differentially affected receptor expression in comparison to their respective lipids A. Free KDO in the equimolar concentration as it exists in KDO2-lipid A (Re-LPS) did not influence the level of CD14 but downregulated the expression of TLR4 and CD11b (Figure 1). Tenfold increased KDO concentration did not affect further the receptor expression. The addition of KDO2 to lipid A *E. coli *- that is, applying KDO as covalently linked constituent of Re-LPS - led to upregulation of CD14 and TLR4 but downregulated CD11b expression. The expression of TLR4 was most pronounced upregulated by Re-LPS *S. enterica *but in the case of CD14 and CD11b this Re-LPS had an opposite effect in comparison to *E. coli *endotoxins (table in Figure 1). Lipid A *S. enterica *was a less potent TNFα inductor than that from *E. coli *(Figure 2). This may be explained by the differences in lipid A composition determining lipid A affinity to target receptor(s). LPS *E. coli*, as had been shown early, caused MyD88-dependent fast NF-κB degradation (rapid TNFα response) whereas LPS *S. enterica *induced MyD88-independent signaling (delayed TNFα response) [5]. In our study, free KDO did not stimulate TNFα release. KDO2 as a constituent of Re-LPS *S. enterica *increased significantly the TNFα-inducing activity of lipid A *S. enterica *but this effect was not so distinguished between Re-LPS *E. coli *and lipid A *E. coli *(Figure 2).

## Conclusion

Free KDO in the used concentration was inactive in regulation of TLR4, CD11b and CD14 expression and did not induce TNFα release but its impact in biological activity was detected when KDO was applied as constituent of Re-LPS. This may be explained by the effect of KDO on the spatial conformation of Re-LPS.
